# Political ecology of milk: Contested futures of a lively food

**DOI:** 10.1111/gec3.12497

**Published:** 2020-06-18

**Authors:** Nathan Clay, Kayla Yurco

**Affiliations:** 1 School of Geography and the Environment, University of Oxford, UK; 2 Geographic Science Program, School of Integrated Sciences, James Madison University, VA

**Keywords:** dairy, development, food studies, food system, livestock, political ecology

## Abstract

This article advances thinking on the political ecology of food and agriculture by reviewing research on milk and dairy. As increasingly contested foods, milk and dairy provide a window onto inter-linked social and environmental crises and attempts to solve them through adjustments to food production and consumption. We critically assess three trajectories of change (*more* milk, *better* milk, and *less* milk) that are representative of broader efforts to fix social-environmental crises through food. Arguing that these efforts eschew systemic change, we discuss how ideas from food studies, agrarian political economy, and development studies can be united in a potentially transformative research agenda on the political ecology of milk (as well as other foods).We reflect on how concepts of justice, power, and care might inform a political ecology of food and agriculture that can help envision and enact more democratic food futures.

## 1 Introduction

*“Rwanda is known as a land of milk and honey, parents should let their children develop a culture of drinking milk, and rearing cattle for it is one of the things that define a true Rwandan.”*—Gérardine Mukeshimana, Rwanda’s Minister of Agriculture and Animal Resources (August 24, 2016).

*“Dairy cows are not gently milked and allowed to roam in the sun and play with friends on factory farms. There is no sunshine or happiness where this milk comes from.”*—Livekindly (2018) blog (January 24).

Contemporary debates about milk are often framed in ways that highlight seemingly intractable, polarizing issues. The above quotes evoke the lively cultural politics of milk at play across the Global North and South. Although they represent different production–consumption systems, such narratives epitomize what DuPuis (2002) has called the “perfect stories” of milk: technology-driven *progress* toward a utopic imaginary of bodily and economic growth, on the one hand; violent, diseased *downfall* of animals, the planet, and human health on the other. These narratives frame problems in binary ways, often pitting rural livelihoods against animal welfare or environmental sustainability against economic growth. Such simplified framings can obscure the complex political ecologies of food production and consumption, serving to entrench rather than transform systemic issues in the contemporary dairy sector (Clay, Garnett, & Lorimer, 2020). This article reviews research on milk and dairy and advances political ecology as a research program that can help identify systemic inequalities and imbalances that underlie dairy’s challenges and opportunities to meeting future nutrition needs while remaining within environmental limits. In so doing, it contributes to recent efforts to link food studies with political ecology (Galt, 2013; Moragues-Faus & Marsden, 2017).

Dairy’s social–environmental intricacies manifest in similar and different ways across the Global North and South. In Europe and North America, growing concern about the environmental and health impacts of animal source foods (FAO, 2006; Springmann, Godfray, Rayner, & Scarborough, 2016; Poore and Nemececk, 2018) has seen dairy emerge as a focal point of environmental politics. A recent front-page article in *The Guardian* entitled “avoiding meat and dairy is the single biggest way to reduce your impact on earth” (Carrington, 2018) drew substantial interest, as did a slew of related articles. Younger consumers (under age 24) are likely to associate dairy with negative environmental impacts (Mintel, 2019). Fluid milk sales in the United States (US) dropped by 8% in 2018 (Dairy Farmers of America, 2019) while sales of plant milk increased by 9% (Plant-Based Foods Association, 2018). This decrease in consumption, together with increasing production costs and consolidation of power in the food system, has resulted in the rapid closure of farms in longstanding dairy regions of the US and European Union (EU) (Blayney, 2002; Jay & Morad, 2007; MacDonald, Jerry, & Roberto, 2016; Hadrich, Wolf, & Johnson, 2017).

Meanwhile, dairy consumption is booming in China and India, where a narrative of milk’s wholesomeness has merged with an agenda of nation building (Ohlan, 2016; Pingali, 2006; Wiley, 2011) that strongly resembles corporate and state efforts to position milk as “nature’s perfect food” a century earlier in the United States (DuPuis, 2002). Dairy continues to be a key source of nutrition and cattle an important store of wealth among rural societies in Africa, Asia, and Latin America, yet access to livestock resources remains highly uneven across class and gender lines (McPeak & Doss, 2006; Clay, 2018; Yurco, 2018). The rapid rise of dairy consumption in the Global South has come alongside food system transformations and the rise of supermarkets, which incorporate small farmers into national and regional markets (Popkin, 2014; Reardon, Peter Timmer, Barrett, & Berdegué, 2003). Dairy commercialization factors prominently in development efforts as a path to reduce malnutrition, lift the rural poor out of poverty, and feed a growing urban middle class (Enahoro, Lannerstad, Pfeifer, & Dominguez-Salas, 2018; FAO, 2018). Yet, these institutional changes have meant rapid consolidation of power in the food system due to high capital requirements to meet safety standards (Farina, Gutman, Lavarello, Nunes, & Reardon, 2005; Reardon & Peter Timmer, 2012); processes that can exclude and marginalize smaller farmers and women (Distefano, 2013; Quisumbing et al., 2015; Reardon, Barret, Berdegue, & Swinnen, 2009; Taverner & Crane, 2018).

In this article, we consider how dairy cuts across issues of environmental sustainability, rural development, animal welfare, and human health. Beneath a sheen of bucolic imagery, the commodification of milk has always been imperfect, forged through complex and sometimes violent relationships among humans, animals, and the environment (DuPuis, 2002; Freidberg, 2009; Gillespie, 2018; Smith-Howard, 2014). We explore these environment–society relationships in the context of broader debates about food, the environment, and social change. A political ecology approach is well-positioned to question environment-society issues surrounding food (Moragues-Faus & Marsden, 2017). Political ecology investigates local–global dynamics to document who wins and who loses amid social–environmental change (Robbins, 2012; Zimmerer & Bassett, 2003) and, more recently, food system transitions (Galt, 2013). While authors have considered the political ecologies of meat (Emel & Neo, 2015; Schneider, 2017), similar work on dairy has been sparse (Gillespie & Collard, 2015). A political ecology approach brings into focus the roots of uneven social–environmental outcomes through place-based assessments that illuminate entrenched patterns of social inequity and ecological degradation. We suggest that a political ecology approach can enhance understanding of food system transformations through central concepts of justice, power, and care.

In the sections that follow, we critically assess three simplified trajectories by which milk is commodified: *more* milk, *better* milk, and *less* milk. We then review research on dairy systems across the Global North and South to identify patterns and gaps in understanding. We draw attention to how social science research on dairy tends to compartmentalize into agrarian political economy, development studies, and critical food studies perspectives. We call for work that unites these perspectives and propose a political ecology research agenda on food system change that advances concepts of justice, power, and care. In conclusion we discuss how food regimes premised on *more, better*, and *less* are contested and relational. We argue that policies and programs rooted in these discrete framings will constrain efforts to transform food systems by enabling the continuation of power imbalances that are firmly embedded within the industrial dairy system.

## 2 The Contested Past and Future of Milk

Milk has long been a substance contested on societal and bodily levels. As with other mammals, humans are generally able to digest milk until 7 years of age, at which point a majority become unable to readily digest milk’s principal sugar, lactose. To counter this, some societies made milk into cheese and yogurt, which are more easily digested because lactose is broken down by bacteria during fermentation (Salque et al., 2013). Around 3,000 to 7,000 years ago, genetic mutations led to the persistence of the lactase enzyme among human populations in East and West Africa, Northern Europe, and parts of Central Asia and the Middle East. These mutations enabled adults to digest lactose and encouraged cultures of rearing animals for milk in these regions (Tishkoff et al., 2007; Leonardi et al., 2012). Dairy’s dietary importance in these societies often led to a veneration for milk as well as the animals producing it and the people caring for them, values that continue to reverberate through contemporary social, political economic, and cultural ecological institutions (Mendelson, 2013; Valenze, 2011; Wiley, 2014). Here we explore these institutions and how they have shaped three overlapping trajectories in the commodification of milk.

Until technologies of pasteurization, transport, and refrigeration, fresh milk was mainly consumed near farms, adulterated with chemicals to make it appear fresh, or made into cheese and yogurt to preserve it (Atkins, 2010; Freidberg, 2009; Mendelson, 2013). In Europe and North America, fresh milk and cheese were commodified in the mid-19th century while agro-industrial dairy systems arose more recently in the Global South. Dairy commodification—that is, the techno-scientific practices and social institutions that standardize production processes and create economic and social value (Bakker, 2007)—is embedded within cultures, political economies, and ecologies (Latour, 1993; Paxson, 2013; Valenze, 2011). Elaborate social-technical regimes emerged in efforts to make milk available year-round and convince consumers that it was safe and nourishing. This necessitated overcoming milk’s material limits: risk of disease, physiological limits of animal bodies, and seasonal constraints on pasture availability (Atkins, 2010; DuPuis, 2002; Freidberg, 2009). Given that human bodies also produce milk, dairy has often been tied to ideals of motherhood, which have been central to milk’s commodification in the North and South (Valenze, 2011; Wiley, 2014). Ironically, demand for milk in the US spiked in the 1920s due in a large part to shifting social norms that reduced the prevalence of breastfeeding (DuPuis, 2002).

By the 20th century, milk became increasingly entangled with narratives of modernization, progress, and nationbuilding in North America and Europe (Atkins, 2010; DuPuis, 2002); a trajectory of commodification defined by a drive to produce *more milk*. Led by a narrative of growing bodies to grow nations (specifically an expanding urban middle class) following World War II, agricultural research and policy created the preconditions for dairy farms in the North to specialize and intensify (Smith-Howard, 2014). Often through colonial and neo-colonial pathways, similar institutions emphasized technology-led productivity increases in Africa, Latin America, and Asia (Clay, 2019; Wiley, 2014). Since the 1950s, milk output in the North has increased exponentially per farm, per cow, and per input of feed and labor. Retailer control of milk supply chains has enabled supermarkets in the North to sell fluid milk at a loss in bids to draw customers in. While this has reduced the retail cost of dairy products, it has also squeezed profit margins on farms, often forcing smaller producers out of business (Clay, Garnett, & Lorimer, 2020; Jay & Morad, 2007). This trend of *more milk* is highly entrenched worldwide (McGregor & Houston, 2017) and continues to underwrite public and private-sector led dairy commercialization in the South, where agroindustry and supermarkets are quickly gaining control of dairy systems (Reardon et al., 2009; Taverner & Crane, 2018). With global milk consumption predicted to increase by 22% over the coming decade as middle classes continue to expand in China, India, Africa, and Latin America (FAO, 2018), this *more* trajectory appears poised to remain the dominant form of milk commodification.

Industrial dairy systems can have negative impacts on the environment, animal welfare, and rural livelihoods (Jay & Morad, 2007; Stuart, Schewe, & Gunderson, 2012; Krieg, 2014; Foote, Joy, & Death, 2015). Recognition of these effects throughout history has continuously led to milks that claim to be *better*. For example, to overcome fears of becoming sick from spoiled milk, “certified” milks emerged in urban North America in the 19th century (Freidberg, 2009). In the 1980s, skimmed milks became popular in the US and EU following health studies that suggested links between dairy consumption and heart disease, a connection that continues to be contested (Dehghan et al., 2018). Organic milk boomed in the US during the 1990s largely as a result of concerns about the use of recombinant bovine growth hormones in industrial dairy production (DuPuis, 2000). Milk alternatives have continued to proliferate over the past decade with myriad offerings now promising to be better for the environment, animal welfare, local economies, and human bodies. In the North, some examples include local, raw, organic, grass-fed, artisanal, fortified, and flavored. In emerging economies, dairy commercialization is similarly premised on notions of betterness. In Brazil and Argentina, for example, public and private milk standards have driven differentiation since the 1990s (Farina et al., 2005). In each of these alternatives to industrial dairy, additional value is sought through the assurance of specific qualities of milk. Little research has considered the processes and impacts of milk alternatives. However, dairy alternatives can mirror the conventionalization seen with other foods that rely upon standards and certification (Guthman, 2004). With local milk, for example, Gupta and Makov (2017) show that inputs originate from far away. Studies show that a prioritization of economies of scale in US organic dairy systems has meant that much organic milk is produced on large farms, sometimes with confined feeding operations that merely substitute organic grain (Diamond, 2013; DuPuis, 2000). These studies raise questions about the degree of systemic transformation that “better” milks can enable.

Plant-based milks (often called mylk, mlk, or m*lk), derived from nuts, grains, legumes, and seeds, have recently boomed in North America and Europe. This third trajectory of milk commodification is premised on an ostensible shift to consuming *less* milk. People are entreated to give up dairy so as to avoid negative environmental, animal welfare, and human health impacts. While soymilk has been consumed in China for hundreds of years, plant milks have boomed in the US and EU over the past decade. The global market doubled from 2010 to 2018, reaching an estimated $16 billion USD (Mintel, 2018), including $1.8 billion in annual sales in the US (Good Food Institute, 2018), or about 15% of the total fluid milk market (Plant-Based Foods Association, 2018). Dairy companies and agro-industry have moved quickly to capitalize on this trend. In 2017, the French dairy giant Danone purchased White Wave/Alpro, the largest plant-milk producer in the world, making Danone the largest supplier of both dairy and plant-based milks. The swiftness with which agro-industry has taken control of the plant milk sector raises questions about the degree of transformation offered by these products, including the claims of environmental sustainability that are often used to market plant milks (Clay et al., 2020). Almond milk, for example, carries forward many of the industrial dynamics that have been the downfall of milk production systems in California (Bladow, 2015). Moreover, even while dairy consumption is dropping in much of the Global North, worldwide dairy consumption is increasing and production is expected to continue increasing apace (FAO, 2018). While the consumption of plant milks constitutes a rejection of exploitative industrial dairy systems that have been continuously propped up by government subsidies (Gambert, 2019), it remains to be seen whether this consumer-led disruption will engender systemic transformation (Clay et al., 2020). Paradoxically, claims of *less* milk might belie an increase in overall milk consumption. With around half of the options on supermarket shelves containing added sugar, the rise of plant milks may be part of an observed global trend of sugar sweetened beverage consumption (Popkin & Hawkes, 2016). Thus, while the *less* milk trajectory promises lower environmental, health, and animal welfare impacts, in practice plant milks have entrenched reliance on agri-industrial systems which come with negative environmental impacts, no guarantee of higher animal welfare in existing industrial livestock systems, and a product that one ultimately needs to consume more of to get the same nutrition as dairy milk (Clay et al., 2020).

## 3 Assessing Milk Regimes

These trajectories of *more*, *better*, and *less* encapsulate current debates about dairy systems. Each milk regime frames problems in ways that privilege certain solutions and actors to implement those solutions. For instance, the intensive dairy systems embedded in a “productivist” regime of *more milk* tend to identify production as the viable realm to solve environmental crises caused by overproduction while holding that it is important to continue producing milk for food security and nutrition. Solutions in the *more* milk trajectory emphasize producers as the actors best positioned to fix environmental problems such as greenhouse gas emissions. These fixes often prioritize increasing efficiency through technological mechanisms (Garnett, 2014). By contrast, the “alternative” regimes of *better* and *less* milk rely almost entirely on consumers to make dietary changes, i.e. buying different products that are seen to provide (or avoid) certain environmental, social, and animal welfare outcomes (Clay, Garnett, & Lorimer, 2020). Yet, milk regimes premised on notions of *better* or *less* may have limited potential to rectify adverse effects associated with industrial dairy production, and can also introduce new challenges to environmental sustainability and equitable rural development (DuPuis, 2000; Diamond, 2013; Gupta, 2016; Bladow, 2015; Gupta & Makov, 2017; Clay et al., 2020). Framing milk futures according to categories of *more, better*, or *less* may therefore have limited transformative potential as these categories fail to acknowledge the overlaps and linkages between these regimes. Importantly, it also discounts the importance of governance changes that may transform relationships between actors rather than rely upon individual producers or consumers. Identifying leverage points to transform how food is produced and consumed necessitates attention to these power dynamics (Galt, 2013; Moragues-Faus & Marsden, 2017; Rossi, Bui, & Marsden, 2019), yet such work is conspicuously lacking for dairy systems (Alston, Clarke, & Whittenbury, 2018; Clay, Garnett, & Lorimer, 2020). Here we build a foundation for this research by reviewing work on the social–environmental dimensions of dairy. We categorize social science research on dairy into agrarian political economy (which often focuses on production in the North), food studies (which tends to emphasize consumption in the North) and development studies (which generally considers production and subsistence consumption in the South). While these categorizations are imperfect, they help us see how seemingly discrete components of dairy systems might be more productively brought together with a political ecology approach.

### 3.1 Agrarian political economy

The political economy of agriculture (or agrarian political economy) investigates how “structural changes” (e.g., policies and markets) in agri-food systems shape the means of production, thereby constraining and/or enabling producers' decision-making (Buttel, 2001). Research on dairy in this vein has illustrated how farms have rapidly consolidated, first in Europe and North America and later in Oceania, Asia, Latin America, and Africa. Structural changes in a dairy sector racing toward efficiency and food safety have resulted in the concentration of power within large farms, processors, and supermarket chains (Hadrich et al., 2017; Jaleta et al., 2013; Jay & Morad, 2007; MacDonald et al., 2016; Pingali, 2006; Wiley, 2011). Agrarian political economy work has demonstrated how an emphasis on productivity has made it challenging for small and medium-size farms to remain viable without making substantial adjustments to farm operations (Blayney, 2002; Davidson & Schwarzweller, 1995; Farina et al., 2005; Jay, 2006; Krieg, 2014). Unceasing price competition is seen to drive changes in traditional skills and ways of life toward models of decision-making that are more in line with corporate worldviews (Davidson, 2002; Diamond, 2013; Jay & Morad, 2007; Taverner & Crane, 2018). Family farms in North America and Europe have given way to scaled-up dairy operations that rely on immigrant labor or robots to do the difficult work of early-morning and late-night milking (Freidberg, 2009; Cricchi, 2017; Butler & Holloway, 2015). As family farming becomes less and less viable in the North, other options for rural employment can also decline and the cultural landscapes of dairy farming often disappear unless they are explicitly subsidized (Flaten, 2002). Yet, a key question in agrarian political economy is also how small farms can persist, which they do in the North through policies that support dairy landscapes for agro-tourism (Freidberg, 2009), value-added products such as artisanal cheese (Paxson, 2013), or unpaid family labor (Alston, Clarke, & Whittenbury, 2017).

Overall, this work has highlighted how the *more milk* regime in the North has promoted overproduction and emphasized profit maximization at the expense of rural livelihoods (Blayney, 2002; Jay, 2006; MacDonald et al., 2016). It has shown how government interventions have both sought to secure access to safe, cheap milk, as well as to protect dairy farmers, often pointing out the complications in reconciling these two aims (DuPuis, 2002; Just, 2009). For example, government regulations such as dairy subsidies and policies like the milk marketing order system in the Northeast US now threaten the small farmers that they may have originally aimed to protect (Freidberg, 2009). This work has demonstrated how market deregulation and corporate control have enabled supermarkets to capture most of the profit in dairy value chains (Collantes, 2019; Moser & Varley, 2013). Agrarian political economy has had limited engagement with dairy in the South (though see, e.g., Farina et al., 2005). However, some have demonstrated North–South linkages such as how the overproduction of milk in the Global North has been continuously channeled to the South as powdered milk, which can undercut prices for locally produced fresh milk and disrupt local economies and nutrition (Freidberg, 2009). Following the repeal of EU milk quotas in 2015, milk powder exports to sub-Saharan Africa tripled, with milk powder selling for a third the cost of fresh milk in West Africa (Choplin, 2016).

### 3.2 Food studies

Work on dairy from critical food studies perspectives often overlaps with agrarian political economy, yet it focuses more on consumption. This work has often taken a historical approach, attending to cultural dynamics of milk production and consumption through poststructural methods (e.g., discourse analysis) that consider how elements of food systems are socially constructed. For example, DuPuis (2002) considers how narratives of efficiency, progress, and bodily health underwrote milk’s rise in the 20th century US. Valenze (2011) demonstrates how government and corporate interventions have sought to position milk as part of a “nutritional social contract.” Mendelson (2013) explores how milk’s cultural prominence enabled the overbreeding of cows and overprocessing of dairy products. Others have discussed how the story of fresh milk is enmeshed in socio-technical arrangements that have driven social change in rural and urban areas of the US and EU (Atkins, 2010; Freidberg, 2009; Martin, 2010). With case studies of 21st century India and China, Wiley (2011, 2014) has demonstrated that notions of affluence and modernity drove recent surges in milk consumption, with the state backing dairy marketing campaigns in efforts to literally grow the nation by nourishing children. Others have provided critiques on the gendered nature of milk’s cultural–political dynamics. Boswell-Penc (2006), for instance, investigates the politics surrounding breastmilk, environmental contaminants in human bodies, and the infant formula industry. Calling for feminist milk studies, Gaard (2013, p. 613) poignantly states that human society has “childishly projected its own gendered image onto nature as selfless and self-sacrificing mother, as in Shel Silverstein’s book *The Giving Tree*, or onto other mammal species, requiring the female bovine to symbolize maternal nature: mindless, patient, slow-moving, lactating.”

Overall, food studies work has demonstrated the fixity of pastoral and motherly imagery across milk regimes, including commodity (Freidberg, 2009; Wiley, 2014) as well as *better* and *less* (Bladow, 2015; DuPuis, 2002). It has also explored how the politics of dairy extend into broader questions about social change. For example, Gupta (2016) illustrates that multiple and conflicting definitions of localness (including how, where, and for whom local milk should be produced) constrain efforts to establish community food systems in Hawai'i. While very little food studies work has considered milk in less industrialized countries, similar questions might be raised about milk’s future in Africa and Latin America. In Rwanda, for example, milk’s social capital flows through gift giving, where it has long been associated with a communal sense of health that has roots in precolonial feudal power dynamics (Taylor, 1992). Through the ongoing *Our Milk, Our Health, Our Future* program, the Rwanda National Dairy Platform is working to link these cultural constructs of milk and reciprocity with the country’s aspirations to achieve middle income country status. As the quote from Rwanda’s Minister of Agriculture and Animal Resources at the start of this article attests, milk’s promise of modernity continues to permeate visions of development.

### 3.3 Development studies

In contrast to food studies' emphasis on the Global North, much of the research on dairy in the South has been from a development studies perspective. In contrast to agrarian political economy’s focus on national-level policies, development studies work is often rooted in cultural ecology (or the local social and environmental components that underpin household decision-making), employing ethnographic methods to evaluate the impacts of development policies and programs on rural and urban livelihoods. Paralleling the growing demand for animal-sourced foods in Asia, Africa, and Latin America (FAO, 2018), public health initiatives and development efforts in these regions continue to emphasize increased consumption of milk to combat malnutrition (Dugdill, Bennett, Phelan, & Scholten, 2013; Enahoro et al., 2018). Milk provides nutritional benefits to poor mothers and children in low and middle-income countries and is particularly essential in pastoralist societies (Grace et al., 2018; Iannotti & Lesorogol, 2014; Sadler, Kerven, Calo, Manske, & Catley, 2009). However, many have raised questions about the ability of development interventions to equitably reduce malnutrition through systems that emulate industrial production in the North. Development programs that aim to transform subsistence dairy systems into more intensive operations can constrain dietary diversity in their emphasis on milk over other staple foods (Aubron, Cochet, Brunschwig, & Moulin, 2009; Ickowitz, Powell, Rowland, Jones, & Sunderland, 2019). Attempts to implement commercial dairying do not automatically confer social equity (Farina et al., 2005). In Africa, such programs have been shown to overlook gender dynamics and the multiple objectives of different actors in dairy value chains (Salmon et al., 2018; Taverner & Crane, 2018). Adherence to a regime of “more milk” (e.g., establishing pasture, importing European cattle breeds, and charging a fee for access) can enable urban elites to benefit while dispossessing subsistence users from pasture access (Clay, 2019).

Overall, this work has suggested that transforming milk regimes through development interventions is seldom straightforward. The complexity and context-specific nature of both development and human nutrition—as well as the high cost of studies considering these dual components—has meant scarce research integrating nutritional evidence with assessments of specific development programs and their impacts on rural livelihoods (Grace et al., 2018; Muehlhoff, Bennett, & McMahon, 2013). This disconnect between studies of nutrition and assessments of development policies may also owe to the fact that research on subsistence livestock systems generally focuses on how livestock are grazed rather than how livestock are managed for milk consumption (Yurco, 2018). The emphasis on grazing management has led to a misguided assumption that development initiatives will improve nutrition if grazing capacity is enhanced (Dugdill et al., 2013; Sadler et al., 2009). In Africa, development policies focusing on grazing are especially problematic as they overlook women’s roles in livestock systems (women most often manage animal health and milk while men attend to grazing duties) and can even create higher labor demands for women (Njuki, Waithanji, Bagalwa, & Kariuki, 2013; Yurco, 2018).

### 3.4 Knowledge gaps

Research on dairy from agrarian political economy, food studies, and development studies perspectives has yielded important insight into how milk production and consumption are embedded in processes of social change. Together, these bodies of work have demonstrated some of the contradictions and social justice implications surrounding the governance of milk in the North and South. However, these three research streams remain largely isolated in their treatment of milk. Most crucially, little attention has been given to how the political and economic interfaces with the cultural and ecological.

There are geographies to this discrepancy. As discussed above, much research on milk in the Global South employs community-based case studies to consider cultural ecology and livelihoods, while research in the North focuses upon broader structural changes and related cultural aspects of milk consumption. In the South, this reinforces the longstanding assumption that food security hinges on development interventions that can increase the availability of food and/or the resources to produce food (Jarosz, 2014). One result of this is that researchers and policymakers overlook the agency and heterogeneity of dairy consumers in the South. On the other hand, the emphasis in agrarian political economy and food studies literature on structural elements and consumption in the North reinforces an erroneous idea that all producers operate in the same way, similarly negating their agency. This makes empirical sense given the prevalence of subsistence modes of milk production–consumption in the South and of agro-industrial modes in the North. However, these simplifications obscure potential alternatives. Without place-based understanding of the diverse lives, livelihoods, and cultural ecologies of milk producers in the North, we are unlikely to shed the imaginary of the dairy industry as a monolithic capitalist operation. Until we see Northern milk producers as heterogeneous, we will likely continue to see consumers and milk companies as the answers to milk’s environmental troubles. Likewise, absent attention to the politics and cultures surrounding milk consumption in the South, development programs will likely continue to see food security as synonymous with the overall production of milk (or even more problematically, of land and cattle).

There are of course exceptions to these rules, some of which we explore below in our suggestions for a research agenda (Section 4). Yet, the overall research predilections script policies in ways that constrain food system transformations. As others have discussed for food systems at large (Galt, 2013; Moragues-Faus & Marsden, 2017), political ecology research offers a way out of this impasse. A political ecology approach considers the interface of structural change (e.g., policies and markets) and the agency of communities (whether they are rural producers or urban consumers) who make decisions within specific cultural, ecological, and social contexts (Robbins, 2012). Through community-based work, political ecology offers a synthesis of political economy and cultural ecology that cuts across the false divide between structure and agency (Zimmerer & Bassett, 2003). As recent work on meat production–consumption demonstrates, political ecology employs case studies of various sites—from farms to government agencies to restaurants to homes—to illuminate how social institutions are built on power imbalances that marginalize certain humans, animals, and ecosystems (Emel, Johnston, & Stoddard, 2015; Emel & Neo, 2015).

We suggest that political ecology work on milk and dairy will help (a) develop contextualized understandings of the politics of human–animal–environment interactions surrounding food production–consumption; (b) investigate how social and interspecies inequities are embedded in animal food systems; and (c) consider past and current trajectories of change in these systems to identify how winners and losers are produced as well as how these patterns are being contested. In the following section, we discuss three interlocking themes that we see as promising for not only identifying obstacles but also for charting opportunities for transformative change in dairy and food systems more broadly: justice, power, and care. These themes bring together insight from feminist perspectives, animal studies, and food studies under an umbrella of political ecology.

## 4 Political Ecologies of Milk: Justice, Power, Care

### 4.1 Justice, gender, and social movements

Food systems worldwide give rise to and reflect a range of environmental and social problems. As discussed above, these issues manifest in the regimes of *more*, *better*, and *less* milk. Political economy and development studies have considered the impacts of structural changes on dairy producers' livelihoods. However, there is much potential to deepen understanding about processes of marginalization in dairy systems through analysis of how power operates in these systems. At the same time, it is essential to consider how social movements (as well as programs and policies) can generate more just and sustainable milk futures.

To investigate marginalization and justice, a political ecology of milk might build on feminist thinking about how the intersections of gender, class, race, and ethnicity can shape experiences (Butler, 1990). Feminist geographers investigate intersectionality by looking to everyday spaces (such as farms, supermarkets, and kitchens) as sites where social identities and power dynamics are renegotiated (Massey, 2005; Elias and Arora-Jonsson, 2017). Despite the fact that around two thirds of the world’s livestock keepers are women, gender and its intersections with other identities has seldom been a focus of work on dairy production and consumption (Wangui, 2014; FAO, 2011). There are a few promising recent studies, however. For example, some have argued that an imaginary of submissive maternal nature normalizes gendered violence against cows (Gaard, 2013; Gillespie, 2018). Alston et al. (2017) demonstrated that the commercialization of family dairy farms in Australia created a commercial-oriented ethos that re-validated men’s roles as farmer-managers while diminishing the value of women’s hidden but equally essential contributions to household labor. As another example, through a feminist ethnographic analysis of a pastoralist community in Kenya, Yurco (2018) discusses how intra-household access to livestock resources is highly uneven and the ways that women use their knowledge of milk and animal health to challenge or even overcome factors that otherwise perpetuate such marginalization.

Alongside this attention to the intricacies of marginalization is the need to re-envision alternatives through engagement with communities and other actors involved in food system transformation (Rossi et al., 2019). Political ecology is considered a community of practice in that the field is defined less by concepts and more by what researchers do and who they work with (Robbins, 2012). Political ecologists studying agri-food often work closely with communities and social movements to consider how processes of marginalization cut across social and environmental dimensions. Such collaborative work is important to ensuring that these movements are not co-opted by agro-industrial food systems (Agyeman & McEntee, 2014). Food justice and agroecology are two movements that hold particular promise for envisioning more equitable and sustainable dairy futures. Food justice aims to identify how social inequalities and processes of marginalization are written into the organization of food production, distribution, and consumption (Alkon & Mares, 2012). In addition to ensuring equitable access to food (Gottlieb & Joshi, 2010), the food justice movement must merge with broader debates and movements against inequality, racism (Herman, Goodman, & Sage, 2018), and animal welfare abuses (Gillespie & Collard, 2015). Agroecology is a field of study as well as a social movement concerned with developing more sustainable and just food systems. It is closely linked with efforts to re-envision food politics and the meaning of citizenship (Patel, 2007) and offers a path for political ecologists to engage their dual commitments of exploring biophysical processes and social justice (Galt, 2013; Vandermeer & Perfecto, 2017). In research on dairy systems, little attention has been given to food justice or agroecology (Clay, Garnett, & Lorimer, 2020; Moraine, Duru, Nicholas, Leterme, & Therond, 2014), yet both offer opportunities for political ecologists to actively engage in processes of social–environmental change.

### 4.2 Power and vitality: Insights from animal geography

Attention to how marginalization is produced and resisted through food production and consumption practices can help challenge the “coercive uniformity” of globalized agri-food systems (McMahon, 2011, p. 409). Ethnographic work can help recast both producers and consumers as complex actors in food system transformations. In considering how social difference and injustice are produced, political ecologists often concentrate on social power and how it circulates through the nonhuman worlds of plants and animals (Bakker & Bridge, 2006; Lave et al., 2014; Sundberg, 2011). Above we alluded to how contemporary dairy landscapes—the rampant farm closures in Europe and North America, the continued hunger in sub-Saharan Africa, the greenhouse gas emissions—are as much the results of agricultural histories, uneven power relations, and class and gender disparities as they are of climatic or ecological processes. The concentration of power in the food industry has led to the systematic exploitation of ecosystems and of animal and human bodies (Boyd et al., 2001; Guthman, 2004; Alkon & Guthman, 2017; Patel & Moore, 2018; Gillespie, 2018).

To explore these ideas further, dairy research can usefully draw from animal studies insights. For one, milk might be considered a lively commodity (Haraway, 2008), its commodification imbued with the physical and symbolic work of humans and animals (Collard & Dempsey, 2013; Porcher & Schmitt, 2012). We suggest that there is scope for research on milk that considers this vitality. Research might consider the human–animal rhythms (Barua, 2016), for example, that make up dairy landscapes, including how livestock are simultaneously commodities and laborers (Barua, 2019) and how the commodification of milk therefore relies on animals' vitality to overcome biological limits and facilitate capital accumulation. As Bakker (2012) suggests of water, we might approach milk’s vitality as the interlocking biophysical, physiological, and ecological processes that contribute to how human societies experience, know, and organize around milk. Milk’s commodification has relied on concealing these processes and the diverse human and nonhuman actors involved (Freidberg, 2009) in how milk *becomes food* (Roe, 2006). Future work might focus on how and by whom these spaces have become hidden. For example, Gillespie (2018) provides ethnographic detail about the hidden places and processes that are essential to the commodification of dairy cow bodies. Narratives about animals in policy documents play a similar role in this sometimes violent obscuring of human–animal relationships (McGregor & Houston, 2017). Even reports that set out to consider the environmental sustainability and climate impact of future production systems tend to “de-animate and render invisible animal bodies” by aggregating animals as units of production and materializing them as protein outputs (Arcari, 2017; p. 69). A similar argument could be made about the common practice in development studies of aggregating ruminant animals into Tropical Livestock Units (TLUs, a simplified metric used to measure a household’s wealth and food availability). TLUs smooth out differences between species, thereby discounting the roles of goats and sheep—which are often of particular importance for women and the poorest households—in grazing ecologies and milk provision (Turner, 2018; Yurco, 2017).

Attention to these various practices of milk commodification is essential if we are to re-politicize the processes surrounding dairy production, distribution, and consumption. Such lines of inquiry raise questions not just about where, but *how* and *why* we are seeing the consolidation of power in dairy systems (in terms of input provision, milk production and processing locations, and retail centers). Political ecologies of milk must not take such economic organization or human–animal relationships as predetermined outcomes of capitalism but as part of broader historically situated social–environmental processes. Such a framing is essential to envisioning alternative economies (Gibson-Graham, 2006) that can balance the multipronged trade-offs of dairy systems—which include consumer health, rural livelihoods, animal welfare, and environmental sustainability (Clay, Garnett, & Lorimer, 2020).

Ethnography (including multispecies ethnographies), participatory research, and close work with communities can help shed light on milk’s vitality and the ways that power dynamics are upheld and contested. Recent studies have shown how the vitality of animals and microbes can synergize with efforts to subvert inequalities, gender roles, and technomanagerial solutions. Paxson’s ethnography of artisanal cheese in the US illustrates how cheese is an “unfinished commodity” (2013, p. 13) because the link between intrinsic values (including livelihoods, sustainable ecologies, and “good” food) and market value are still being worked out. This partial commodification stands in marked contrast to the attempts to equate milk with perfection in the 20th century US (DuPuis, 2002). Importantly, the unfinished nature of artisanal cheese creates a substrate for struggles over meanings to play out (Paxson, 2013). As another example, Yurco (2017) investigates clandestine milk trading operations among marginalized women in Maasai households, who employ their intimate knowledge and their role as milkers to exercise control over resources. As a third example, technologies such as robotic milking machines promise to enhance animal welfare and alleviate farm labor burdens by enabling cows to be milked whenever they choose. However, these machines are also demonstrated to constrict animal movement by inscribing activities in ways consistent with zero grazing, by not enabling all cows to benefit equally (some cows do not trust the machines), and by reducing workers' experiential understanding of animals' needs (Butler & Holloway, 2015; Holloway, Bear, & Wilkinson, 2014). In each of these cases, multiple actors (including animals) vie to negotiate the micropolitics of dairy systems. Within such “lively livelihoods” (Emel et al., 2015) lies the possibility for transformation: for humans and animals to work together to challenge coercive power dynamics. Future work might consider how animals' “atmospheres”—that is, how their presences are shaped by and shape their surroundings, including humans and the environment (Lorimer, Hodgetts, & Barua, 2019)—can direct and inhibit various forms of dairy commodification as well as alternative human-animal-environment interactions.

### 4.3 Milk’s care: Biopower and responsibility

As discussed above, questions about milk are often framed in terms of ethics. Does dairy production impinge on animal rights or is it an important source of nutrition? Does milk production exacerbate global warming and environmental destruction or is it an essential livelihood? These framings are saturated with biopower (i.e., who gets to decide whose life matters?) and responsibility (i.e., who is to blame and whose job is it to solve these issues?). Biopower and responsibility are not static. As Paxson (2013) shows, dairy systems conjure multiple values and moralities that may shift over time in response to social, political, and cultural change (Paxson, 2013). In 19th century America, milk filled a gap as a food for infants due to insufficient calorie intake among lower class women (due to poverty) and among upper class women (due to a romantic aesthetic of bodily form that prized dainty eating) (DuPuis, 2002, p. 53). Indeed, valuation (a key step in commodification) requires abstraction and standardization—processes that can silence or hide practices of food production, including the animal and human (often female) bodies producing food (Gillespie, 2018; McMahon, 2011). Standards such as pasteurization sanitize elements of production practices in ways that often obscure the continued violence of agroindustry (Freidberg, 2009; Miele, 2011) while conditioning consumers to not only accept ultra-processed food but to feel good about it (Carolan, 2011). Such erasures underscore the biopower of milk, or the discursive and material processes by which milk (and milk consumers) are made viable and constructed as “good” or “bad.”

Tracing biopower in food systems allows us to see how producers and consumers are “responsibilized” to care in ways that call upon them to make “better” individual choices (Lavis, 2015). Embedding quality markers for foods and eaters in the social consciousness legitimizes certain modes of production and consumption in ways that can recreate and intensify gender, class, and racial inequities (Guthman, 2012). For instance, women and ethnic minorities are often the targets of public food safety campaigns, a practice that makes them “scapegoats for broader socioenvironmental problems,” thereby burying options for needed systemic change (Mansfield, 2012, p. 589). In short, managing food—a metabolically essential input for bodies and cultures alike—is often part of broader strategies and agendas of managing life (Nally, 2011). Food’s biopolitics are diffuse: the assemblages that give rise to “quality" are finely networked through broader socio-cultural values and institutions. Qualities such as freshness are enacted through assemblages that includes producers, supermarkets, and consumers (Jackson, Evans, Truninger, Meah, & Baptista, 2018). This makes the creation of “ethical foodscapes” through market mechanisms an inherently slippery and value-laden process, full of politics and biases (Goodman, 2010). Yet, such agendas can be contested. For example, Kurtz, Trauger, and Passidomo (2013) discuss how raw milk regulation in Georgia acts as a form of biopower that is contested through alternative food networks. Attention to these oppositions at multiple sites and among various groups of actors can yield crucial understanding about clandestine power dynamics, illuminating leverage points to make food systems more democratic (Guthman, 2011; Sexton, 2018).

Making food systems more democratic also necessitates moving past polarizing narratives about the goods and bads of milk. Future work on dairy might do this through attention to a “practical ethics of care” (Puig de la Bellacasa, 2017) that, for example, producers exercise in their everyday work with animals and that inform consumers' decisions about what milk to buy or not. A practical ethics of care forces us to consider the emotional nature of food and the practices of its production and consumption. How we feel about food is arguably inseparable from such “embodied practices, socio-institutional arrangements, and cultural conventions whence it came” (Carolan, 2015, p. 319). Creating space and time for care is particularly important at this juncture of the globalized agrifood system, wherein opportunities for relating to others through caring are conspicuously far-flung and yet the unease of this distance has simultaneously been finessed through certifications such as fair trade in order to commodify notions of consumer care (Goodman, 2004). As Puig de la Bellacasa (2015) demonstrates with soil fertility maintenance through an ethnographic approach on the practical ethic of care, there are practices by which people enact multiple cares (including for animals, their family, their community, or the global environment). This plurality of cares is important. For example, the productivist logics of industrial dairy production can lead to violent forms of care (Overstreet, 2018). Recognizing the complexities of and pluralities of care can be particularly powerful to generate transformations in the deeply situated and complex human and nonhuman assemblages of milk production and consumption. This requires place-based research that is the bread and butter of political ecology.

## 5 Practicing Care in Political Ecology of Food

The future role of livestock in sustainable and equitable food systems is contested and uncertain. In dairy systems, the overwhelming drive to increase efficiency has had disastrous effects on the environment and animal welfare (Foote et al., 2015; Jay & Morad, 2007; Stuart et al., 2012). Rapid consolidation in the dairy industry has consistently entrenched inequities between rural and urban people (Clay, Garnett, & Lorimer, 2020; Freidberg, 2009; Hadrich et al., 2017; Krieg, 2014). This article has reviewed how these effects emerge in complex ways that are embedded in places. We have argued that the debates about milk (often premised on notions of *more, better*, or *less*) tend to ignore these complexities. The recent Twitter campaigns of Veganuary (a month of eating vegan) and Februdairy (the dairy industry’s rebuttal with a month celebrating milk) (Sandhu, 2019) provide a window onto the divisiveness and polarized notions of care in the ongoing debate about milk and dairy. Vegan activists posted evidence of animal welfare abuses to attest to their care for cows and calves and to justify their calls to remove animals from food systems. Dairy farmers posted images of healthy cows as evidence of their daily practices of care for animals while arguing that vegans care little about farmers' livelihoods. The rise of plant-based milks and a social media landscape that facilitates virtue-signaling about food consumption choices makes it even easier for these groups to continue talking past each other (Clay et al., 2020).

Milk futures premised on *more, better*, or *less* do not address these plural notions of care. Nor do they acknowledge how humans' very existence is predicated upon interrelationships with nonhumans: animals, soil, water. Meanwhile, dairy production continues to intensify in Europe and North America as well as in China and India. Platforms such as sustainable intensification, organic production, and plant milks offer limited engagement with the complex social–environmental dynamics of dairy systems (Clay, Garnett, & Lorimer, 2020; Garnett, 2014; Röös et al., 2017). These platforms are often pinned to outcomes defined rigidly as *more, better*, or *less* such that the rich histories of dairy landscapes are erased. The current infatuation with data-driven global-level studies employing life cycle assessments to compare the environmental footprints of various foods similarly obfuscates the social and environmental heterogeneity within and across dairy landscapes. Political ecological work—grounded in place and time—provides a countercurrent to these erasures. This work can inform a more practical ethic of care that is essential for human–nonhuman interdependence to flourish (Puig de la Bellacasa, 2010). It can also speak to the coexistence of multiple cares (be they about rural livelihoods, climate change, food security, and animal welfare).

In these ways, political ecological work is essential to visualizing and empowering transformations in dairy and broader food systems. As a community of practice that is often embedded in processes of social change (Robbins, 2012; Galt, 2013), political ecology can also play a vital role in transformation. To do so it must both critically analyze and participate in movements. These dual practices require engaged fieldwork (Puig de la Bellacasa, 2011). In terms of dairy systems, this means devoting curiosity to human–animal relationships—in all their material and discursive complexity—as some have recently done (Gillespie, 2018; Paxson, 2013). Attention to power, justice, and care (as discussed above) through a political ecology lens will help document winners and losers (both human and animal) in various dairy presents and futures. Practicing care in the political ecology of food might also entail cultivating experiences that bring people together to engage with food in non-commercial spaces, in creative ways that encourage people to reflect on their relationships to the environment, animals, and to other humans (Roe and Buser (2016). Engaged awareness of such complex interconnections may help generate acceptance of imperfect milk stories (DuPuis, 2002) and an ethic of care that is situated in daily practices. Such stories could help speak to food policies and also cultivate shared responsibility for the myriad imperfections that are generated through our species' efforts to feed itself.
